# Accelerating the Adoption of Best Practice Research in Resuscitation Through Implementation Science: Identifying Gaps and Pathways

**DOI:** 10.3390/jcm15020648

**Published:** 2026-01-14

**Authors:** Shohreh Majd, Sze Ling Chan, Mojca Bizjak-Mikic, Marcus E. H. Ong

**Affiliations:** 1 Council of Ambulance Authorities, Hilton, SA 5033, Australia; 2 College of Medicine and Public Health, Flinders University, Bedford Park, SA 5042, Australia; 3 Health Services Research Centre, SingHealth, Singapore 169856, Singapore; 4 Health Services Research and Population Health, Duke-NUS Medical School, Singapore 169857, Singapore; 5 Department of Emergency Medicine, Singapore General Hospital, Singapore 169608, Singapore

**Keywords:** resuscitation, implementation science, knowledge-to-action, healthcare delivery, patient outcomes

## Abstract

Translation of evidence-based resuscitation practices into clinical settings remains slow and inconsistent, a gap that significantly impacts survival and neurological outcomes. Implementation science offers a structured approach to accelerate adoption by identifying context-specific barriers—such as dispatcher workload, team choreography, and resource constraints—and tailoring strategies to overcome them. This paper applies the Knowledge-to-Action (KTA) framework to resuscitation, emphasizing stakeholder engagement, iterative monitoring, and sustainability. We provide detailed guidance across key resuscitation settings, including dispatch-assisted cardiopulmonary resuscitation (DA-CPR), in-hospital code teams, and emergency medical services (EMS). The manuscript introduces a comprehensive outcomes framework encompassing implementation, service/system, and patient-level metrics, and illustrates practical application through case examples such as DA-CPR and real-time feedback devices. To enhance scientific utility, we also present a decision-oriented table for pilot testing, offering healthcare institutions a roadmap for sustainable integration of evidence-based resuscitation protocols.

## 1. Introduction

Effective resuscitation practices play a pivotal role in mitigating the adverse consequences of critical medical situations, ultimately contributing to improved patient outcomes. Despite the availability of robust, evidence-based guidelines, their consistent implementation in clinical practice remains uneven, exposing patients to variations in care quality [1]. Numerous challenges hinder the effective translation of best practice guidelines into actionable strategies within healthcare systems, despite decades of research and evidence generation [2]. This persistent gap between evidence and practice underscores the necessity of adopting systematic implementation approaches [3]. In the context of resuscitation, significant strides have been made in the formulation of best practice recommendations [4]. However, the development of such guidelines is only the first step, while their impact is largely dependent on successful and sustained integration into everyday clinical workflows [5]. Unfortunately, the path from guideline creation to broad-scale adoption remains slow and inconsistent, and this highlights the need for targeted strategies to facilitate uptake [6].

The ongoing disconnect between research evidence and real-world clinical practice in resuscitation highlights the essential role of implementation science (IS) in closing this divide. As a multidisciplinary field, IS systematically examines the processes, contextual factors, and strategies involved in translating research into practice [6,7]. It addresses key questions around how and why implementation succeeds or fails, and it equips stakeholders with practical tools to support the uptake of best practices. For example, frameworks such as the Knowledge-to-Action (KTA) Framework [8] and the Consolidated Framework for Implementation Research (CFIR) [9] provide structured approaches to guide the implementation process. These models emphasise assessing barriers and facilitators, engaging stakeholders, tailoring interventions, and monitoring progress. Importantly, IS promotes a collaborative, systems-level approach that involves researchers, healthcare professionals, implementation scientists, and decision-makers working together to co-design, adapt, and embed evidence-based interventions into everyday care. In the context of resuscitation, applying such frameworks can accelerate the integration of proven practices, thereby improving outcomes and enhancing the quality of patient care [10].

**Figure 1:**
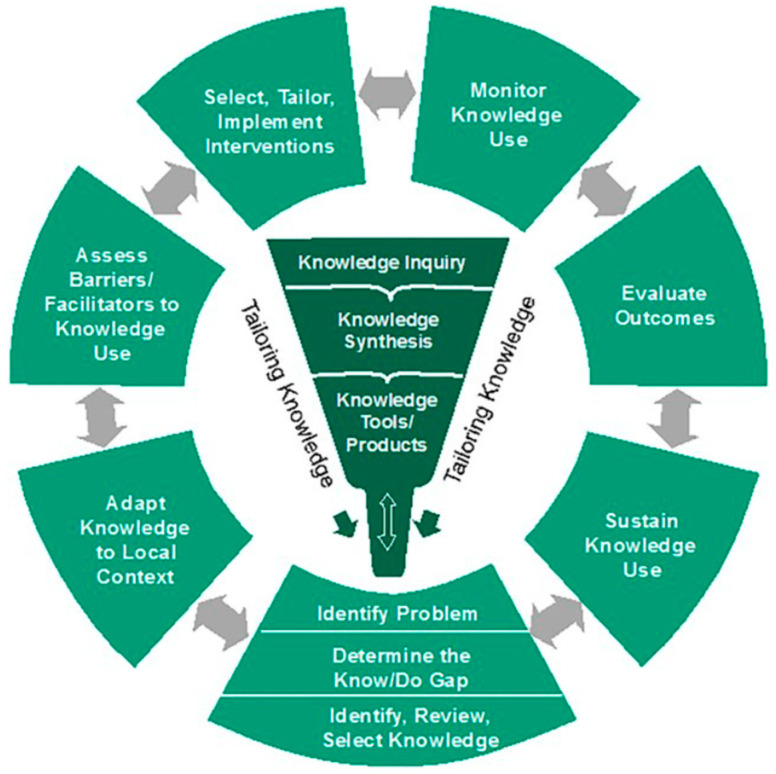
Knowledge To Action (KTA) Framework: The KTA process consists of 2 concepts: knowledge creation and action, with the ideal phases of each concept depicted in the figure. The boundaries between the 2 concepts are not fixed and can influence each other. The action phases can also be performed simultaneously or sequentially, and can be iterative, as depicted by the double-ended arrows. Image from Vogel JP. et al. (2016) [8].

Over time, the collaborative efforts of multidisciplinary stakeholders such as clinicians, researchers, implementation scientists, and policymakers have generated invaluable insights into the challenges and nuances of implementing evidence-based resuscitation practices. These insights are grounded in decades of practical experience and scholarly work by leaders in resuscitation science and implementation research. In this commentary, we synthesise these high-level insights and present them using the KTA framework (Figure 1), a well-established conceptual model that describes the dynamic process of translating knowledge into clinical application [8]. This serves to demonstrate the utility of theoretical frameworks within the IS toolbox to systematically guide and evaluate implementation processes. Furthermore, we use this framework to highlight key research gaps in the adoption of resuscitation best practices and present two illustrative case examples of real-world implementation of specific resuscitation interventions.

## 2. Methodology

This work is a narrative review supplemented by expert commentary. We searched PubMed, Scopus, and Google Scholar using keywords such as “resuscitation,” “implementation science,” “CPR adoption,” and “knowledge translation.” Articles published in English within the last 10 years were prioritised, alongside seminal works on implementation frameworks. We synthesised findings using the KTA framework to structure barriers, strategies, and outcomes.

## 3. Insights from Implementation of Resuscitation Best Practices

### 3.1. KTA: Knowledge Creation

**Figure 2:**
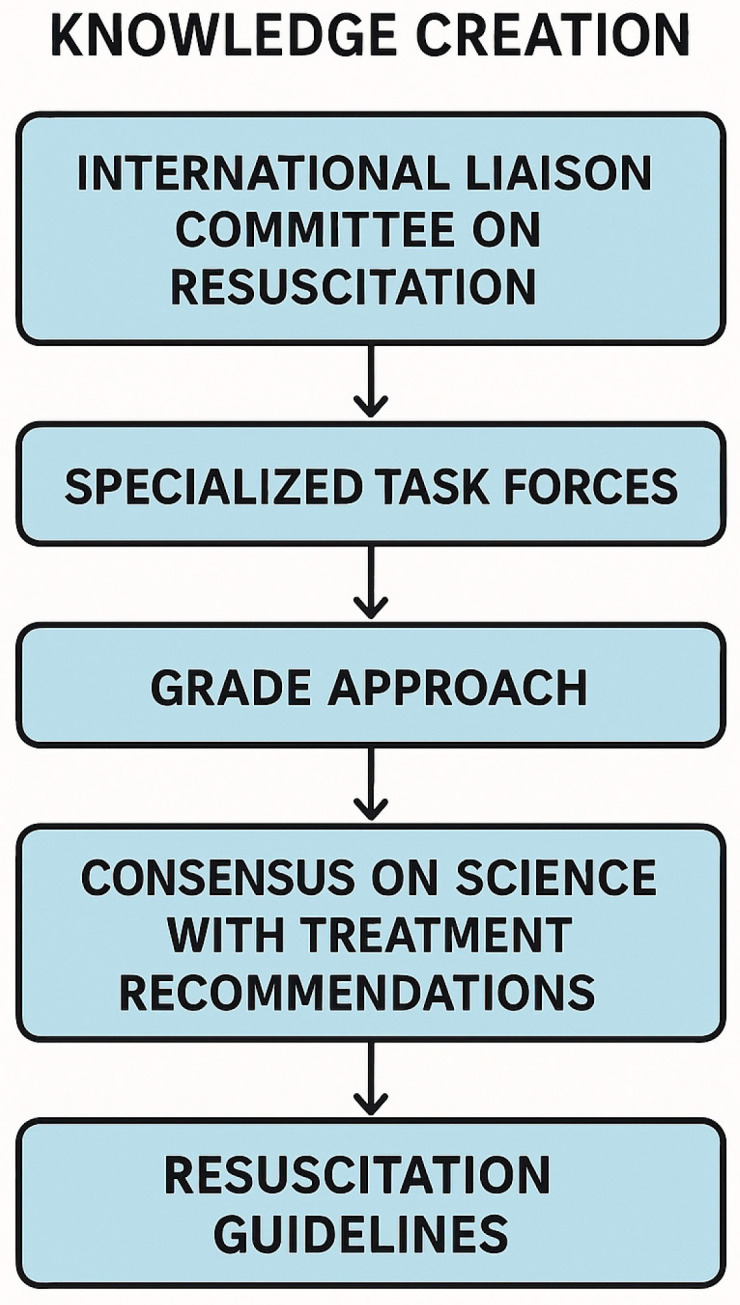
Knowledge creation process in evidence-based resuscitation, from ILCOR’s evidence review to CoSTRs and the development of resuscitation guidelines.

Evidence-based medicine is deeply embedded in the field of resuscitation, underpinned by a structured and iterative process of knowledge creation. This process is led by the International Liaison Committee on Resuscitation (ILCOR), a global consortium of resuscitation councils that systematically evaluates emerging scientific evidence to inform clinical practice. ILCOR operates through specialised task forces that continuously review resuscitation literature using rigorous methodologies, such as the GRADE (Grading of Recommendations Assessment, Development and Evaluation) approach, to assess the certainty of evidence and formulate treatment recommendations. These evaluations culminate in Consensus on Science with Treatment Recommendations (CoSTRs), which are updated regularly and serve as the foundation for resuscitation guidelines developed by regional member organisations, such as the American Heart Association and the European Resuscitation Council. This collaborative and transparent approach ensures that the resulting guidelines reflect the most current, high-quality evidence, while also addressing clinical relevance and feasibility in practice [11,12]. This process is summarised in Figure 2, which illustrates the pathway from evidence review by ILCOR to the development of resuscitation guidelines.

To facilitate consistent data reporting and enable comparison across systems, the Utstein-style reporting template was developed. It provides standardised definitions and structure for collecting and reporting cardiac arrest data, enhancing the quality and comparability of resuscitation research and outcomes [11,13].

In parallel, the Global Resuscitation Alliance (GRA) has proposed a set of ten implementation-focused steps to improve survival after cardiac arrest. Initially developed for out-of-hospital cardiac arrest (OHCA), these steps have recently been adapted for in-hospital cardiac arrest (IHCA). The framework emphasises continuous quality improvement, strong clinical leadership, education, and data use to drive improved survival outcomes [14,15].

Despite these developments, a persistent challenge of translating evidence-based recommendations into routine clinical practice has remained. IS provides a structured approach to address this gap by identifying barriers and facilitators, testing strategies, and evaluating outcomes to ensure sustainable practice change [16].

### 3.2. KTA: Action Cycle

#### 3.2.1. Identify Problem

**Figure 3:**
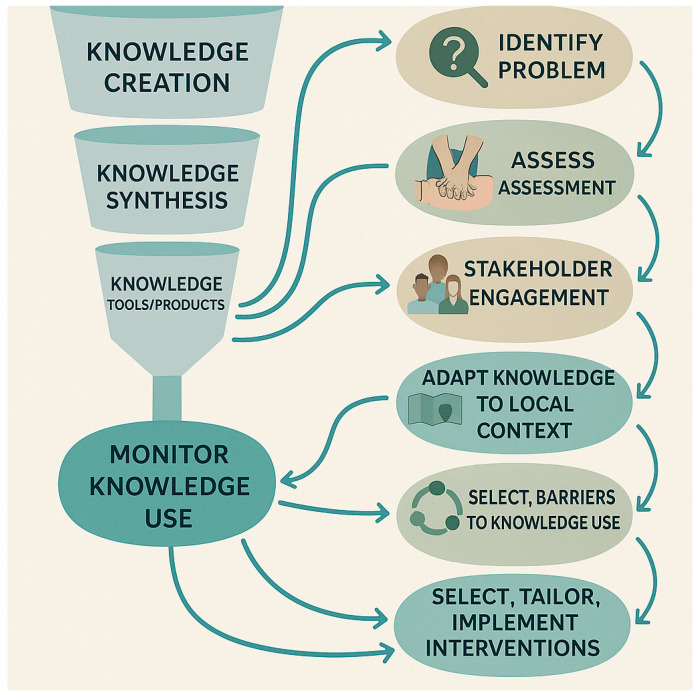
Adapted Knowledge-to-Action (KTA) Cycle for Resuscitation: The key steps for translating evidence into resuscitation practice: knowledge creation, contextual adaptation, implementation, evaluation, and ongoing refinement and responsiveness.

Gaps in resuscitation best practices occur at various stages of implementation across different settings. Some environments struggle with adoption, others with execution, and some with scaling and sustainability. Bridging the divide between research evidence and clinical practice requires a systematic and context-sensitive approach. Figure 3 presents an enhanced version of the KTA framework, tailored to support the implementation of resuscitation evidence. This adapted model illustrates how knowledge translation processes can be systematically applied to improve resuscitation outcomes through iterative, evidence-informed, and locally adaptable strategies

##### Assessment

The initial step involves a comprehensive assessment of existing resuscitation protocols within each healthcare setting. This evaluation should encompass response times, availability and functionality of equipment, and staff proficiency levels. Adherence to established guidelines, such as those provided by the Resuscitation Council UK, is crucial to identify areas requiring improvement [17]. Engaging multidisciplinary teams, including resuscitation personnel, emergency department staff, and quality improvement experts, ensures a holistic understanding of current practices and facilitates the development of targeted intervention strategies [18].

High-quality cardiopulmonary resuscitation (CPR) is essential for improving resuscitation outcomes, and training and feedback mechanisms play a significant role in enhancing CPR performance. Structured training sessions combined with real-time feedback have been shown to significantly improve the quality of chest compressions and overall CPR effectiveness. Regular, hands-on practice supported by immediate performance feedback enhances both the technical proficiency and the confidence of healthcare providers, ultimately preparing them to perform resuscitation more effectively in real-world situations [19].

Figure 3 (adapted KTA cycle for resuscitation; original schematic created by the authors) shows the modifications from standard KTA: (1) explicit stakeholder engagement throughout; (2) strengthened iterative monitoring and feedback loops using registry, device, and dispatch data; (3) emphasis on sustainability and scale-up planning; and (4) resuscitation-specific examples embedded at each step. These modifications are necessary due to the time-critical nature of resuscitation, team choreography requirements, and prehospital–hospital interfaces.

Worked Example: Mapping Dispatcher-Assisted CPR Across KTA Steps

**Identifying the Problem**

In the context of OHCA, the primary problem is the low rate of bystander CPR and the delayed initiation of chest compressions. These issues contribute significantly to poor patient outcomes, making it crucial to address them through effective interventions that can increase the speed and efficiency of the resuscitation process.

**Adapting Knowledge to the Local Context**

To address these challenges, the introduction of standardised dispatcher-assisted CPR (DA-CPR) protocols and training is essential. This adaptation ensures that dispatchers are well-equipped to provide real-time guidance to bystanders, enabling them to perform CPR correctly and effectively until professional medical assistance arrives.

**Assessing Barriers**

Several barriers must be assessed when implementing DA-CPR. These include dispatcher workload, variability in the protocols used by different dispatch centers, delayed recognition of OHCA, and issues related to public awareness and willingness to perform CPR. These barriers must be carefully considered to design effective strategies that overcome them.

**Selecting, Tailoring, and Implementing the Strategy**

A structured approach to implementing DA-CPR involves several key components. First, comprehensive training programs should be developed, including both didactic sessions and simulation-based training, to prepare dispatchers for real-world scenarios. Regular quality assurance (QA) audits of call recordings should be conducted to ensure that DA-CPR protocols are followed properly. In addition, public education campaigns aimed at improving OHCA recognition and increasing the willingness of bystanders to perform CPR should be launched. Key performance indicators (KPIs) should also be set, such as diagnosing OHCA within one minute and initiating DA-CPR within three minutes, to ensure that the response is timely and effective.

**Deploying Multifaceted Strategies Across Levels**

The successful deployment of DA-CPR requires a multifaceted approach. Education and training should focus on simulation-based, low-dose, high-frequency methods to ensure that dispatchers receive continuous practice. Stakeholder engagement is critical, involving clinicians, dispatchers, middle managers, patients, and the public to ensure the implementation is supported at all levels. Interdisciplinary collaboration should be encouraged to bring together professionals from diverse fields, ensuring a well-rounded approach to the intervention. Resource mapping, including the use of process mapping and Failure Mode and Effects Analysis (FMEA), will help identify gaps in resources and optimise their use. Finally, adaptive implementation should be employed, allowing for iterative learning and adjustments based on feedback from all stakeholders.

**Monitoring Knowledge Use**

Monitoring the use of DA-CPR protocols is essential for ensuring the intervention’s effectiveness. Regular audits of DA-CPR call recordings, using standardised assessment tools, will help track compliance. Key timestamps such as call receipt, OHCA recognition, and instruction initiation should be recorded, along with data from registry fields related to bystander CPR. Technology-enabled feedback systems, including dashboards and electronic health record (EHR) analytics, can be used to track adoption and assess the impact of the program. Suggested metrics for monitoring include adoption rates (the percentage of dispatchers using the DA-CPR protocol), fidelity (adherence to script guidelines and CPR feedback device usage), feasibility (training completion rates and workflow integration), acceptability (feedback from staff and patients), penetration (coverage across units and services), and sustainability (long-term adherence to DA-CPR practices). These metrics should be gathered from data sources such as Utstein registry fields, dispatch timestamps, defibrillator downloads, and CPR feedback device logs.

**Evaluating Outcomes**

To evaluate the success of DA-CPR implementation, several outcomes should be measured. These include:

- Adoption: The percentage of dispatchers using the DA-CPR protocol.
- Fidelity: The degree of adherence to the scripted protocol during calls.
- Service Outcomes: Metrics such as time to first compression, CPR quality (rate, depth, recoil), and time to defibrillation.
- Patient Outcomes: Survival rates, neurological status (e.g., Glasgow Coma Scale or cerebral performance category (CPC) score), and Return of Spontaneous Circulation (ROSC).

**Sustaining Knowledge Use**

Sustaining the use of DA-CPR protocols is critical for long-term success. This requires ongoing refresher training and regular feedback provided during shift rounds. Continuous QA processes should be maintained, and public education campaigns should be updated to keep OHCA recognition and bystander CPR top of mind. To scale successful interventions, comprehensive implementation plans must be developed. These plans should outline timelines, roles, and communication strategies, and ensure the integration of ongoing training, fidelity monitoring, and periodic evaluation. Leveraging existing infrastructure and quality improvement systems will help sustain the improvements achieved through DA-CPR, ensuring that these practices remain in place across departments and care settings over time.

##### Stakeholder Engagement

Establishing robust partnerships with key stakeholders develops a collaborative environment essential for successful implementation. Involving resuscitation teams, physicians, nurses, paramedics, administrators, and quality improvement specialists from the outset encourages active participation and ensures diverse perspectives are considered. Open lines of communication facilitate ongoing dialogue and feedback exchange, empowering stakeholders to take ownership of the implementation process and promoting a collective commitment to change [20]. Table 1 explains the implementation determinants and strategies by resuscitation setting, providing a detailed overview of the key barriers and strategies specific to dispatch-assisted CPR, in-hospital code teams, and emergency medical services (EMS) systems.

Effective stakeholder engagement involves identifying key stakeholders, understanding their interests and concerns, and actively involving them in decision-making processes. This approach not only enhances the relevance and acceptability of interventions but also builds trust and collaboration among all parties involved. For example, Stelfox et al. (2015) suggested that engaging a diverse group of stakeholders in critical care settings led to the identification of priorities for improving the quality and value of healthcare [21]. In another recent study, Barnes-Harris and her team investigated the importance of multidisciplinary support and a realistic understanding of health status in motivating engagement in CPR discussions [22]. Their study found that clinicians who had access to multidisciplinary support, including training and preparation, were better equipped to conduct effective CPR discussions. This support provided both physical and psychological capability, enabling clinicians to approach these conversations with confidence and clarity. Furthermore, a realistic understanding of the patient’s health status and the likely outcomes of CPR was crucial for all stakeholders, including patients and their families. This understanding helped to set appropriate expectations and facilitated more meaningful and informed discussions about resuscitation preferences. Through ensuring that all parties have a clear and realistic view of the potential benefits and limitations of CPR, healthcare providers can adopt a more collaborative and supportive environment for decision-making [22].

##### Setting Clear Goals

Defining clear, measurable, and attainable goals aligned with the overarching aim of enhancing resuscitation practices is vital. These goals should directly address specific challenges identified during the assessment phase. Employing the SMART criteria of Specific, Measurable, Achievable, Relevant, and Time-bound ensures that objectives are well-structured and attainable. Effective communication of these goals to all stakeholders is another factor that guarantees the effectiveness of such collaborative efforts in achieving desired outcomes [1].

Setting clear goals involves not only defining the desired outcomes but also outlining the steps needed to achieve them. This process should include regular monitoring and evaluation to track progress and make necessary adjustments. Regular monitoring allows for the identification of any deviations from the planned course of action and provides opportunities to implement corrective measures promptly. Evaluation, on the other hand, helps assess the effectiveness of interventions and ensures that goals remain relevant and achievable over time. Achieving informed goal setting in resuscitation decision-making requires an understanding of patient, provider, and system factors, each with its own priorities. This also involves identifying key barriers and facilitators that influence resuscitation decisions, such as patient health status, provider expertise, and system-level constraints [3].

#### 3.2.2. Adapt Knowledge to Local Context

Tailoring interventions to align with the unique needs and constraints of each healthcare institution enhances the efficiency and effectiveness of the implementation process. Rather than adopting all best practices simultaneously, institutions should strategically select interventions with the greatest potential impact and adaptability to available resources. For instance, implementing high-performance CPR protocols, conducting regular simulation training, or refining communication strategies during resuscitation can be prioritised based on their feasibility and compatibility with existing workflows [8,23]. Engaging stakeholders in decision-making processes ensures alignment with organisational priorities and secures buy-in for the selected interventions.

#### 3.2.3. Assess Barriers to Knowledge Use

Different healthcare settings face barriers at each step of the implementation process, from adoption to implementation and finally scaling up. These barriers can be categorised into three main domains: knowledge barriers, individual barriers, and organisational barriers.

##### Knowledge Barriers

A significant barrier to adopting best practices in resuscitation is the lack of awareness among clinicians regarding updated guidelines and the rationale behind them. Misconceptions about the effectiveness of current practices can lead to resistance to change. In some cases, healthcare professionals may not be adequately informed about the latest advancements in resuscitation techniques or may hold onto outdated beliefs about the efficacy of certain interventions. Addressing these barriers requires comprehensive education and training initiatives to ensure clinicians are up to date with the latest evidence-based guidelines and understand the rationale behind recommended practices. Providing clear, evidence-based information can help dispel misconceptions and garner support for adopting best practices [8].

##### Individual Barriers

At the individual level, several well-documented barriers can impede the adoption of best practice resuscitation guidelines. These include resistance to change driven by entrenched clinical habits, fear of the unknown, and concerns about increased workload or disruption to workflow [24,25]. Healthcare professionals may be reluctant to deviate from familiar routines or may question the clinical effectiveness or safety of newly introduced practices. Perceptions of increased complexity, lack of confidence in new skills, or concerns about time constraints can further exacerbate resistance [26].

These individual-level barriers are not unique to resuscitation but are commonly identified in broader the IS literature. They can be conceptualised using existing behaviour change and implementation frameworks, such as the Theoretical Domains Framework (TDF), which maps determinants of behaviour to specific domains like knowledge, beliefs about capabilities, emotions, and environmental context [27]. Incorporating such frameworks enables a structured assessment of personal and contextual factors influencing practitioner behaviour.

To support the adoption of new practices, targeted interventions may include promoting a culture that embraces change, offering peer support and mentorship, providing comprehensive training, and addressing workload-related concerns to safeguard job satisfaction and reduce burnout. A conceptual diagram summarising individual-level barriers and aligned intervention strategies, such as motivation, skill development, and environmental restructuring, could help visually clarify this relationship for implementation planning.

##### Organisational Barriers

Healthcare systems often face challenges related to resource constraints, lack of standardised protocols, and inefficient communication between different departments, all of which suspend the integration of best practice into resuscitation protocols. Resource constraints, such as limited funding or staffing shortages, can impede the implementation of new practices. These constraints encompass various aspects, including financial limitations, personnel shortages, technological deficiencies, equipment inadequacies, and physical infrastructure constraints. In healthcare settings, introducing state-of-the-art resuscitation techniques often entails specialised training for staff, acquisition of advanced equipment, and facility modifications, all of which demand additional resources. Particularly for institutions grappling with tight budgets and staffing shortages, these demands can pose substantial burdens [28].

Even when successful implementation occurs in one setting, scaling up can be impeded by similar resource limitations and the absence of standardised protocols, leading to inconsistencies in care delivery across different healthcare environments [29]. Inefficient communication between departments further exacerbates these challenges, resulting in fragmented care and missed opportunities for improvement. It has been demonstrated that the lack of a clear implementation plan and insufficient leadership support can stall efforts to change established practices [30].

#### 3.2.4. Select, Tailor, Implement Interventions

Implementing evidence-based resuscitation practices necessitates a multifaceted approach targeting various healthcare system levels tailored to addressing the barriers outlined earlier. Here are some strategies for overcoming the above-mentioned barriers that are likely applicable to most settings.

##### Education and Training

Comprehensive education and training initiatives are vital for equipping healthcare professionals with the necessary knowledge and skills to adopt evidence-based guidelines. Beyond information dissemination, training programs should focus on the practical application of new techniques through hands-on simulations and case studies. Ongoing educational opportunities and refresher courses ensure that clinicians remain current with evolving best practices in resuscitation [31]. Effective education and training programs should incorporate several key elements to maximise their impact. The simulation-based training has been shown to significantly improve the proficiency of healthcare providers in performing resuscitation techniques. High-fidelity simulations that mimic real-life scenarios allow practitioners to practice and refine their skills in a controlled environment, leading to better preparedness and performance during actual resuscitation events [3].

##### Stakeholder Engagement

Continual stakeholder engagement throughout the implementation process is essential to achieving sustained adoption of best practices in resuscitation. Involving frontline clinicians, administrators, and patients in decision-making processes helps develop a sense of ownership and accountability, which enhances motivation and adherence to new practices. Collaborative stakeholder efforts often generate innovative solutions to practical barriers such as resource constraints, including strategies like equipment sharing or optimising workforce deployment [20,32].

There are multiple groups of key stakeholders who influence the implementation of resuscitation best practices. These include healthcare providers, hospital and emergency service administrators, policy makers, educators, researchers, and professional organisations. The public, especially as potential bystanders in OHCA scenarios, is also a critical stakeholder group. Public engagement through community education, awareness campaigns, and initiatives such as CPR training programs has been shown to significantly improve survival rates by increasing early intervention rates prior to professional care [33,34]. Recognising the public as both beneficiaries and active participants in the chain of survival highlights the need for their inclusion in planning and implementation strategies.

##### Clinicians and Paramedics

Securing clinician buy-in is paramount for successful implementation. Providing clinicians with sufficient time to understand the rationale behind new practices and offering opportunities for input significantly enhances buy-in. Incorporating clinicians’ insights and feedback during the planning stages allows for the customisation of implementation strategies to address specific concerns and preferences. Rushing the adoption process may lead to resistance and scepticism, which results in suboptimal outcomes [28].

##### Middle Management

Involving middle managers in the initial planning stages of adopting best practice research in resuscitation provides a significant advantage. These managers possess valuable insights into the operational intricacies of healthcare delivery, making them instrumental in crafting practical implementation strategies. Engaging middle management early ensures the allocation of necessary resources, support, and attention from higher leadership levels. This early involvement creates a sense of ownership and accountability throughout the implementation journey [30].

##### Leadership

Effective leadership is instrumental in driving organisational change towards evidence-based resuscitation practices. Leaders should actively champion the adoption of best practices, articulating a compelling vision for improvement and providing clear directions to staff. Promoting a supportive environment and strategically allocating resources assist leaders in empowering frontline clinicians to embrace change and contribute to the implementation process. Moreover, leadership engagement extends beyond top-level executives to include middle managers who play a crucial role in translating vision into action at the operational level [28].

##### Interdisciplinary Collaboration

Encouraging collaboration across different healthcare departments creates a more integrated approach to resuscitation care. Establishing multidisciplinary teams allows diverse perspectives and expertise to converge, enriching decision-making processes and elevating innovation. Regular interdisciplinary training sessions facilitate knowledge exchange and cultivate a culture of mutual respect and shared accountability among healthcare professionals [31].

##### Resource Mapping

Conducting resource mapping involves assessing existing resources within an institution and identifying gaps that may impede the implementation process. This strategic assessment enables healthcare leaders to allocate resources more effectively, ensuring essential components like training, equipment, and infrastructure are adequately provided for. For instance, if budget constraints hinder the acquisition of advanced resuscitation equipment, resource mapping may reveal opportunities for reallocating funds or establishing external partnerships. The decision-making frameworks poses a high importance in priority setting and resource allocation, which can guide the effective resource mapping process [35].

In an article published in 2024, Eric Roseen discussed the use of process mapping with failure mode and effects analysis (FMEA) to identify determinants of implementation. This method combines qualitative and quantitative approaches to develop process maps and evaluate them for potential failures. Systematic application of FMEA will enable healthcare institutions to prioritise barriers and devise strategies to address them, providing a structured approach to resource assessment [36].

##### Adaptive Implementation Strategies

Recognising the nuanced challenges inherent in healthcare settings, healthcare organisations must adopt implementation strategies to suit local contexts. This involves conducting thorough assessments of organisational culture, workflow dynamics, and resource availability to inform the design of targeted interventions. Embracing flexibility allows healthcare institutions to adapt their approaches based on emerging evidence, stakeholder feedback, and contextual factors [5]. Developing a continuous learning mindset and remaining open to experimentation, will be a strong tool in the hands of healthcare system to refine the implementation efforts iteratively and optimise outcomes over time [37].

##### Pilot Testing

Before full-scale implementation, it is crucial to conduct thorough pilot testing of selected interventions in a controlled environment. Simulation labs or designated resuscitation areas provide an effective setting to simulate real-world scenarios, helping to identify potential barriers, refine protocols, and train staff in a low-risk environment. Pilot testing plays a vital role in refining interventions, as highlighted by studies on feasibility and pilot studies [38]. Additionally, the use of mixed methods in pilot feasibility studies helps inform the design and implementation of intervention trials, allowing for valuable feedback from participants and stakeholders to enhance intervention strategies [39]. These understandings are essential for fine-tuning protocols and addressing unanticipated challenges before broader implementation.

Table 2 summarises the pilot testing objectives, measures, and decision thresholds for scaling up CPR. Three pilot tests proposed to evaluate the implementation of updated resuscitation protocols, focusing on simulation-based assessment, training effectiveness, and feasibility in real emergency settings.

##### Formation of Implementation Teams

Establishing multidisciplinary implementation teams and developing cross-functional collaboration are essential elements for connecting diverse expertise and perspectives that drive implementation efforts forward. Formalising roles within these teams ensures dedicated focus and accountability, as was shown by McGuier [40] and her team. Their work also highlighted the importance of teamwork in healthcare innovation and emphasised the advantages of multidisciplinary collaboration. Open and ongoing communication between team members supports dynamic knowledge exchange. This approach enables researchers to gain insights into real-world implementation challenges, while helping practitioners develop a deeper understanding of the evidence underpinning new practices.

#### 3.2.5. Monitor Knowledge Use

Collecting and analysing data on the adoption and impact of new practices is essential for guiding decision-making and continuous improvement. Beyond basic metrics such as compliance rates, healthcare organisations should prioritise gathering insights from frontline clinicians and patients to understand the lived experiences and perspectives surrounding resuscitation care. Harnessing technology-enabled feedback mechanisms, such as real-time dashboards and electronic health record analytics, facilitates timely identification of implementation barriers and enables proactive intervention. Transparently sharing performance data with stakeholders cultivates a culture of accountability and empowers frontline staff to drive quality improvement initiatives collaboratively. Establishing clear communication channels and providing ongoing support to frontline staff improves a culture of continuous improvement and innovation [41].

Gathering feedback from healthcare providers and patients aids in identifying adoption barriers and enablers. This iterative approach facilitates real-time strategy adjustments, ensuring alignment with evolving healthcare system needs. Establishing robust data collection mechanisms and leveraging technology to track implementation progress can provide valuable insights for decision-making and optimisation. In addition, promoting a culture of transparency and accountability where feedback is acted upon can enhance stakeholder engagement and drive continuous improvement efforts [42].

In the setting of resuscitation implementation, it is crucial to collect and analyse data on both adoption and impact using technology-enabled feedback systems, such as dashboards and EHR analytics. Suggested measures include adoption, which can be assessed by the percentage of DA-CPR protocol usage; fidelity, which involves adherence to protocol scripts and utilisation of CPR feedback devices; feasibility, which encompasses training completion and workflow integration; acceptability, based on feedback from staff and patients; penetration, determined by the coverage across units or services; and sustainability, which refers to the maintenance of practices over time. Data sources for these measures include Utstein registry fields, dispatch timestamps, defibrillator downloads, CPR feedback device logs, and EHR event markers.

#### 3.2.6. Evaluate Outcomes

Creating mechanisms for ongoing evaluation and feedback is essential to continuously monitor the impact of implemented practices on patient outcomes and resuscitation performance. Gathering data on KPIs, including survival rates, neurological outcomes, and adherence to protocols, is crucial. Regularly reviewing performance metrics and engaging stakeholders in discussions to identify areas for improvement are critical components of this process. Utilising real-time data and feedback to refine implementation strategies, optimise protocols, and enhance resuscitation processes over time is essential. Cultivating a culture of continuous learning and improvement is fundamental for sustaining success in resuscitation practices [43,44].

The framework for outcomes in resuscitation implementation includes:

- Implementation outcomes: adoption, fidelity, feasibility, acceptability, penetration, sustainability. Examples: % of dispatchers using DA-CPR protocol; adherence to CPR feedback device use.
- Service/system outcomes: response times, CPR quality metrics (rate, depth, recoil, pauses), time-to-defibrillation, team coordination indicators.
- Patient outcomes: survival to discharge, neurological status (e.g., CPC score), ROSC rates.

Measurement should align with data sources available (registry fields, device logs, dispatch/EHR timestamps), and be reported transparently to enable learning and benchmarking.

#### 3.2.7. Sustain Knowledge Use

After successful pilot testing, it is crucial to expand interventions to cover the entire healthcare setting. This expansion necessitates the development of a comprehensive implementation plan, detailing timelines, roles, and communication strategies. This involves conducting thorough contextual analyses and pilot studies to assess intervention feasibility across varied settings. Gradual scaling, coupled with continuous outcome evaluation, facilitates adjustments and optimisation of implementation strategies. Leveraging existing infrastructure and building upon successful pilot initiatives can streamline the scaling process and enhance sustainability. Equally important is ensuring that staff across departments and care settings receive adequate training and support. Considering factors like workflow integration, resource allocation, and sustainability is vital for ensuring successful and widespread adoption. Monitoring implementation fidelity and making necessary adjustments to maintain consistency and effectiveness throughout the organisation are also paramount [45,46].

## 4. Opportunities in Resuscitation Implementation

### 4.1. Leveraging Implementation Scientists’ Expertise

Implementation scientists play a key role in bridging the gap between research and practice. Their expertise in translating research into real-world settings can significantly enhance the success of adoption efforts. Collaboration with implementation scientists enables healthcare organisations to design evidence-based and contextually relevant implementation strategies. These experts identify barriers, develop tailored interventions, and offer ongoing guidance throughout the implementation process, ensuring a structured, data-driven approach that yields efficient and effective results [47].

### 4.2. Building Capacity Through Implementation Science Training

IS underscores the need to identify specific skills critical for intervention execution. This encompasses not only technical skills but also adaptive ones like effective communication and leadership. Investing in training programs to equip healthcare professionals with the requisite skills for implementing new resuscitation techniques effectively is essential. Healthcare professionals and administrators involved in implementing new resuscitation practices necessitate a diverse skill set encompassing change management, communication, and data analysis. However, the dynamic nature of healthcare mandates a comprehensive understanding of both technical and adaptive skills, which may not always be clearly delineated. Tailored training programs addressing these skill gaps enhance healthcare providers’ readiness for change. Promoting a learning culture where feedback is valued, and professional development opportunities are readily accessible can advance skill development and build organisational capacity for successful implementation [3].

Investing in training programs in IS principles empowers healthcare professionals across various roles to drive successful implementation. Equipped with knowledge in IS, clinicians, middle managers, and administrators become effective change agents within their spheres of influence. Training covers essential topics such as readiness assessment, designing implementation plans, progress measurement, and feedback adaptation. Nurturing a cadre of implementation-savvy professionals will assist health organisations promote a culture of continuous improvement and innovation in resuscitation practices [48].

### 4.3. Promoting Diversity in Implementation Teams

Diversity within implementation teams enriches the planning and execution of adoption strategies. Team members from diverse clinical backgrounds, specialties, cultural contexts, and functional roles contribute unique perspectives that help identify challenges and generate innovative solutions. A diverse implementation team enhances engagement with various stakeholders, ensuring that the implementation strategy is comprehensive, inclusive, and sensitive to the needs of all involved parties. Ultimately, this leads to more successful and sustainable adoption outcomes [40,49].

## 5. Case Studies

### 5.1. Implementation of Real-Time CPR Feedback Devices at the Children’s Hospital at Westmead (CHW), Australia

The Children’s Hospital at Westmead in Sydney implemented real-time CPR feedback devices in their Paediatric Intensive Care Unit (PICU) to improve CPR quality during IHCA. A multidisciplinary team, including clinicians, educators, and administrators, developed a training program integrating simulation and performance feedback. The quality of CPR significantly improved, with target-compliant compressions rising from 20.7% to 74.8% [50]. This case illustrates the effectiveness of diverse implementation teams in integrating technological innovation.

### 5.2. Collaborative Efforts to Improve Cardiac Arrest Survival Across Australia and New Zealand

In 2018, a Resuscitation Academy event united leaders from EMS in Australia and New Zealand. Agencies like St John WA and the South Australian Ambulance Service co-developed high-performance CPR (HP-CPR) models, adapting them to local needs through shared strategies and ongoing inter-agency communication. This cross-organisational collaboration underscores the value of stakeholder diversity in resuscitation implementation [51].

### 5.3. Establishment of the Aus-ROC Epistry for Out-of-Hospital Cardiac Arrest (OHCA)

The Australian Resuscitation Outcomes Consortium (Aus-ROC) established a transnational OHCA registry, “the Epistry”, which brought together EMS agencies, academic researchers, and hospitals. Through harmonising data collection and sharing, this diverse group enabled a deeper understanding of OHCA outcomes and informed practice improvements across the region [52].

### 5.4. Implementation of Dispatcher-Assisted CPR in Asia

DA-CPR is recognised as one of the “Ten Steps to Improve Survival” by the GRA, given its proven capacity to increase bystander CPR rates and improve outcomes for OHCA. A key initiative in this area is the Pan Asian Resuscitation Outcomes Study (PAROS), which conducted a large-scale, population-based implementation trial across Asia from 2009 to 2018. This trial evaluated the effectiveness of a comprehensive DA-CPR package across 33 sites in countries including Japan, Korea, Singapore, Malaysia, and others, collectively covering a population of approximately 165.4 million. The study demonstrated that structured implementation of DA-CPR, including dispatcher training, protocol standardisation, and quality improvement measures, led to significant increases in bystander CPR rates and improvements in patient outcomes [53].

The implementation package included introduction and adoption of a standardised DA-CPR protocol and training package which included didactic lectures, small group discussions, online training and weekly in-service small group sessions with practical scenarios. Sites adopting the comprehensive package were required to collect dispatch recordings of DA-CPR and use a standardised audit tool that captured timings, recognition of OHCA, quality of telephone instructions and barriers to CPR. The audit data was given as feedback to individual dispatchers and discussed at shift rounds to reinforce learning. Specific KPIs were identified including being able to diagnose OHCA within one minute and to start DA-CPR within 3 min. A public education program was introduced across implementation sites to improve recognition of OHCA and enhance collaboration with emergency dispatchers. The trial enrolled 170,687 cases, and a before-and-after comparison revealed improved survival to hospital discharge during the implementation period across all groups. The comprehensive intervention group showed significantly higher odds of receiving bystander CPR and achieving survival with good neurological outcomes compared to the control group [53].

## 6. Conclusions

The integration of IS into resuscitation practice enhances patient outcomes and strengthens healthcare delivery. Addressing known barriers, engaging key stakeholders early, including clinicians, middle managers, and implementation scientists, and promoting diversity within implementation teams increase the likelihood of successful adoption of evidence-based practices. Beyond reiterating the need for education, this paper offers a practical roadmap for implementation: integrating DA-CPR protocols, leveraging real-time CPR feedback devices, and using registry-driven monitoring to ensure fidelity and sustainability. These strategies, grounded in IS, enable healthcare systems to overcome entrenched barriers and achieve measurable improvements in survival and neurological outcomes.

## 7. Limitation

Our synthesis may reflect sampling bias due to the predominance of studies from high-income countries. Financial constraints and cost-effectiveness considerations were not deeply explored, despite their potential impact on implementation success. Additionally, unsuccessful implementation cases were underrepresented in the literature, limiting discussion of failure modes.

## Figures and Tables

**Table 1:** Implementation determinants and strategies by resuscitation setting.

Setting	Key Barriers	Implementation Strategies	Monitoring Metrics
Dispatch-assisted CPR	Public awareness; dispatcher workload; protocol variability; timely OHCA recognition	Standardised scripts; structured training and simulations; QA audits of call recordings; public campaigns	Call audits and timestamps; DA-CPR initiation time; registry fields (bystander CPR, time intervals)
In-hospital code teams	Team choreography; equipment readiness; role clarity; leadership engagement	In situ simulations; pre-assigned roles; leadership-supported debriefing; real-time CPR feedback devices	EHR event logs; CPR feedback device metrics; adherence to team protocol
EMS systems	Prehospital–hospital interfaces; resource constraints; protocol variation; data reporting	Interagency protocols; HP-CPR; feedback devices; early defibrillation; regional QA/benchmarking	Utstein registry fields; defibrillator downloads; response/transport times

**Table 2:** Pilot test objectives, measures, and decision thresholds for scaling up interventions in resuscitation settings.

Pilot Objective	Measures	Decision Threshold for Scale-up
Simulation-based assessment	CPR quality scores; team coordination; scenario timing	≥90% protocol compliance across scenarios
Training effectiveness	Pre/post knowledge tests; skills retention at 3–6 months	≥80% improvement post-training and ≥70% retention
Feasibility in emergency care	Time-to-defibrillation; staff feedback; workflow fit	≥80% positive feasibility rating and no adverse workflow impact

## Data Availability

The original contributions presented in this study are included in the article. As this is a review article, no new data were generated or analyzed during the study. Therefore, there are no publicly available datasets associated with this work. Further inquiries regarding the data or methodology can be directed to the corresponding authors.
